# Circ-malat1 promotes gastric cell growth via miR-154-5p/CCND2

**DOI:** 10.3389/fgene.2025.1594354

**Published:** 2025-07-10

**Authors:** Lina Li, Liuqing Yang, Yuzhen Wang, Kejun Nan

**Affiliations:** ^1^ Department of Oncology, The First Affiliated Hospital of Xi’an Jiaotong University, Xi’an, Shaanxi, China; ^2^ Department of Oncology, Shaanxi Provincial Tumor Hospital, Xi’an, Shaanxi, China; ^3^ Department of Digestive, Second Affiliated Hospital of Xi’an Medical University, Xi’an, Shaanxi, China

**Keywords:** Circ-malat1, gastric cancer, cell growth, miR-154-5p, CCND2

## Abstract

Recent studies have shown that circular RNA (circRNA) plays an important role in the development of gastric cancer. However, despite the widespread use of high-throughput sequencing technologies, the function of many circRNAs in gastric cancer remains unclear. In this study, we selected a circ_0002082 (circ-malat1) that is differentially expressed between normal gastric epithelial cells and gastric cancer cells to further investigate its role and molecular mechanisms in regulating gastric cancer development. The study primarily explores the function and molecular mechanisms of circ-malat1 at the cellular and molecular levels. Functional studies reveal that overexpression of circ-malat1 promotes gastric cancer cell growth. Conversely, silencing the expression of circ-malat1 has the opposite effect. Mechanistic studies indicate that circ-malat1 is predominantly expressed in the cytoplasm of gastric cancer cells and can act as a competing endogenous RNA by sequestering miR-154-5p, thereby enhancing CCND2 gene expression. In conclusion, circ-malat1 promotes the development of gastric cancer by competitively binding to miR-154-5p. Based on literature reports, it is speculated that circ-malat1 may also participate in regulation through other pathways, which require further investigation.

## Introduction

Gastric cancer can be classified into several types based on various factors including histology (cellular characteristics), genotypes, and molecular features (Hu et al., 2012). Among these factors, genetic factors play a significant role in gastric tumorigenesis (Resende et al., 2010). The genetic regulation of gastric tumorigenesis involves multiple levels, including gene expression, protein function, and cellular signaling (Molaei et al., 2018). In gastric tumorigenesis, abnormal gene expression may lead to uncontrolled growth of tumor cells. Understanding the features of gene regulation may help in the development of new therapeutic targets for gastric cancer (Sutter and Fechner, 2006). Recently, researchers have primarily focused on identifying and regulating these abnormal gene expressions to inhibit the growth of gastric cancer cells. Non-coding RNAs (ncRNAs) play a crucial role in the occurrence of gastric cancer (Xie et al., 2020). These ncRNAs include microRNAs (miRNAs) (Liu and Xiao, 2014), long non-coding RNAs (lncRNAs) (Tan et al., 2020), and circular RNAs (circRNAs) (Li et al., 2020), participating in the regulation of various biological processes, including cell cycle, proliferation, apoptosis, migration, and invasion.

CircRNAs are a type of non-coding RNA that forms a covalently closed-loop structure, which does not have 5′or 3′ends and are mainly produced by variable processing of precursor mRNAs (pre-mRNAs) (Ebbesen et al., 2017). Because circRNAs lack free ends, they are resistant to degradation by exonucleases, which makes them more stable (Jeck et al., 2013). In recent years, with the development of RNA high-throughput sequencing technology, more and more circular RNAs have been screened and identified (Jiao et al., 2021). CircRNAs have been found to play an important role in the development of several diseases, including tumors and carcinogenesis. CircRNAs, as a type of regulators, can be used as prognostic indicators or targets to predict or treat cancer in the future, and its importance cannot be ignored (Xu et al., 2020). In gastric cancer, some researchers have reported the function of circRNAs. These functional circular RNAs regulate gastric cancer through various mechanisms, including miRNA sponge (Lin et al., 2020), translation Function (Jiang et al., 2021), and protein binding (Ma et al., 2022). Circular RNA YAP1 functions as a tumor suppressor in gastric cancer cells by sponging miR-367-5p, thereby promoting the expression of p27 ^Kip1^ (Liu et al., 2018). Circular RNA EIF4G3 is downregulated in gastric cancer and suppresses tumor growth and metastasis through the β-catenin signaling pathway. The study found that circEIF4G3 binds to the δ-catenin protein, promoting its TRIM25-mediated ubiquitin degradation (Zang et al., 2022). circMAPK1 (hsa_circ_0004872) was also found to be downregulated in gastric cancer tissues. This circRNA encodes a novel protein consisting of 109 amino acids, and the inhibition of tumorigenesis is associated with the expression of this protein (Jiang et al., 2021). Despite studies investigating the regulation of circRNA in gastric cancer, the functions of numerous circRNAs in this context remain unknown.

Herein, the expression of circ_0002082 (circ-malat1) was found to differ between normal gastric epithelial cells and gastric cancer cells, which captured our attention. This study aims to explore the molecular mechanisms by which circ-malat1 regulates gastric carcinogenesis at both cellular and molecular levels. Specifically, we examine the role of circ-malat1 in modulating the growth of gastric cancer cells. Our findings suggest that circ-malat1 may function as a competitive endogenous RNA, exerting regulatory influence over these processes. These results provide a theoretical foundation for further research on the role of circular RNAs in the development of gastric cancer.

## Materials and methods

### Cell culture

GES-1 cell line was supplied by the Cell Bank of Chinese Academy of Sciences. BGC-823 cell line was purchased from Beyotime. They were cultured in RPMI-1640 medium (Hyclone), supplemented with 10% fetal bovine serum (Gibco), 0.1 mg/mL streptomycin, and 100 U/mL penicillin. All these cells were incubated at 37°C with a 5% CO_2_ concentration.

### Plasmid construction, small RNA synthesis, and cell transfection

The full-length sequence of circ-malat1 was amplified by PCR and inserted into the circular RNA expression vector pcD25-ciR. The 3′UTR (wild type or mutant) sequence of the CCND2 gene and the sequence of circ-malat1 (wild type or mutant) were also amplified and inserted into the dual-luciferase reporter vector psi-check2, respectively. Circ-malat1 siRNA, miR-154-5p mimic, and inhibitor were synthesized by Genepharma. After the vector construction is completed or the small RNA is synthesized, the cells are transfected using Lipofectamine 2000 (Invitrogen), following the procedure outlined in the instructions.

### RNA extraction and real-time quantitative PCR

Total cellular RNA was extracted using Trizol reagent (Vazyme) following the manufacturer’s instructions. Subsequently, 1 μg of total RNA, either treated with RNase R (Epicenter Technologies) or untreated, was reverse transcribed into cDNA using the PrimeScript™ FAST RT reagent Kit with gDNA Eraser (Takara), following the manufacturer’s instructions. qRT-PCR was performed using SYBR Green (Vazyme) on an ABI qPCR system, and fold changes were determined using the relative quantification 2^−ΔΔCT^ method. Additionally, the nuclear and cytoplasmic fractions of cells were separated using the PARIS™ Kit (Invitrogen™) following the manufacturer’s instructions. Expression ratios of specific RNA molecules between the nuclear and cytoplasmic fractions were subsequently assessed by qRT-PCR. The primer sequences used are listed in Supplementary Table S1.

### Western blot

RIPA lysis buffer containing PMSF and protease inhibitors was added to the cells, and the protein concentration was measured using a BCA kit (Beyotime). Equal amounts of total protein were loaded into each well for SDS-PAGE gel electrophoresis, followed by transfer to a methanol-activated PVDF membrane. After blocking with skim milk at room temperature for 1 hour, the membrane was incubated with the corresponding primary antibody overnight at a low temperature. The membrane was then incubated with the appropriate secondary antibody for 1 hour at room temperature and developed using an ECL luminescent solution (Beyotime). The antibody information is listed in Supplementary Table S2.

### RNA immunoprecipitation

RNA immunoprecipitation was performed according to the protocol provided with the Geneseed kit. Briefly, cells were lysed using a solution containing protease inhibitors and RNase inhibitors. The lysate was incubated for 10 min, followed by low-temperature centrifugation to obtain the supernatant. A 50 μL aliquot of the supernatant was reserved as the input group, mixed with loading buffer, boiled, and stored at low temperature for subsequent protein immunoblotting. An additional 100 μL of the supernatant was taken directly for RNA extraction. Next, 200 μL of protein A + G beads were washed three times with buffer, thoroughly mixed with fresh buffer, then divided into two equal parts, with Ago2 antibody added to one part and IgG antibody added to the other. The mixtures were rotated at low temperatures for 2 h, followed by multiple washes, discarding the supernatant each time. Afterward, the Ago2 and IgG antibody-bound beads were incubated with fresh lysis solution and rotated slowly at low temperature overnight. Using a magnetic stand, the supernatant was discarded, and the beads were washed three times with washing solution. In the final wash, 100 μL of the magnetic bead complex was collected, placed on the magnetic stand, and the supernatant was discarded. Loading buffer was added to the beads, boiled, and placed back on the magnetic stand, and the supernatant was collected for protein immunoblotting analysis. The remaining magnetic bead complex was used for RNA extraction, followed by qRT-PCR detection.

### CCK-8 assay

The growth status of BGC-823 cells was measured by a CCK-8 kit (Solarbio) following the manufacturer’s instructions. In brief, the cells were seeded on the 96 well plates. After transfection for about 24 h, add 10 μL CCK-8 reagent to each well and continue to incubate for 4 h. To detect the absorbance value at 450 nm by using a microplate reader (Tecan).

### EdU assay

Seed 3,000 cells in the growth phase per well into a 96-well plate. Twenty hours after transfection, perform the following operations according to the EDU kit instructions (RiboBio). Briefly, add EdU-containing medium (1:1,000) and continue culturing for 4 hours. Then, fix the cells with a solution containing 4% paraformaldehyde for 30 min. Stain the cells with Apollo staining solution for 30 min, and after washing, stain the nuclei with Hoechst 33,342 staining solution for 10 min. Finally, observe the cells under a fluorescence microscope.

### Cell cycle assay

Inoculate the counted cells into a 60 mm culture dish. Once the cells have adhered and grown stably, further process them according to experimental requirements. When the cell density reaches approximately 80%, collect and fix the cells using a PBS solution containing 70% ethanol. The cell cycle is then analyzed by flow cytometry (BD Biosciences) according to the instructions provided with the cell cycle kit (MedChemExpress, HY-K1071).

### Cell apoptosis assay

The counted cells were seeded into a 6-well plate. After the cells had grown stably, they were processed according to the experimental requirements. The adherent cells were then digested with EDTA-free trypsin and washed with PBS. Subsequently, follow the instructions of the apoptosis kit (MultiSciences, AP101) to analyze cell apoptosis using flow cytometry.

### Dual-luciferase reporter assay

Wild-type or mutant circ-malat1 (or CCND2) dual-luciferase reporter vectors were co-transfected with miR-154-5p mimics in HEK293T cells, respectively. Twenty-four hours after transfection, luciferase activity was analyzed using a dual-luciferase reporter assay kit (Beyotime), following the manufacturer’s instructions. Firefly luciferase activity served as the control.

### Statistical analysis

The data in the experiment are presented as means ± SEM. Significance was analyzed using t-tests with GraphPad Prism software (*p < 0.05; **p < 0.01).

## Results

### Expression characteristics of circ-malat1 in gastric cancer cells

**FIGURE 1:**
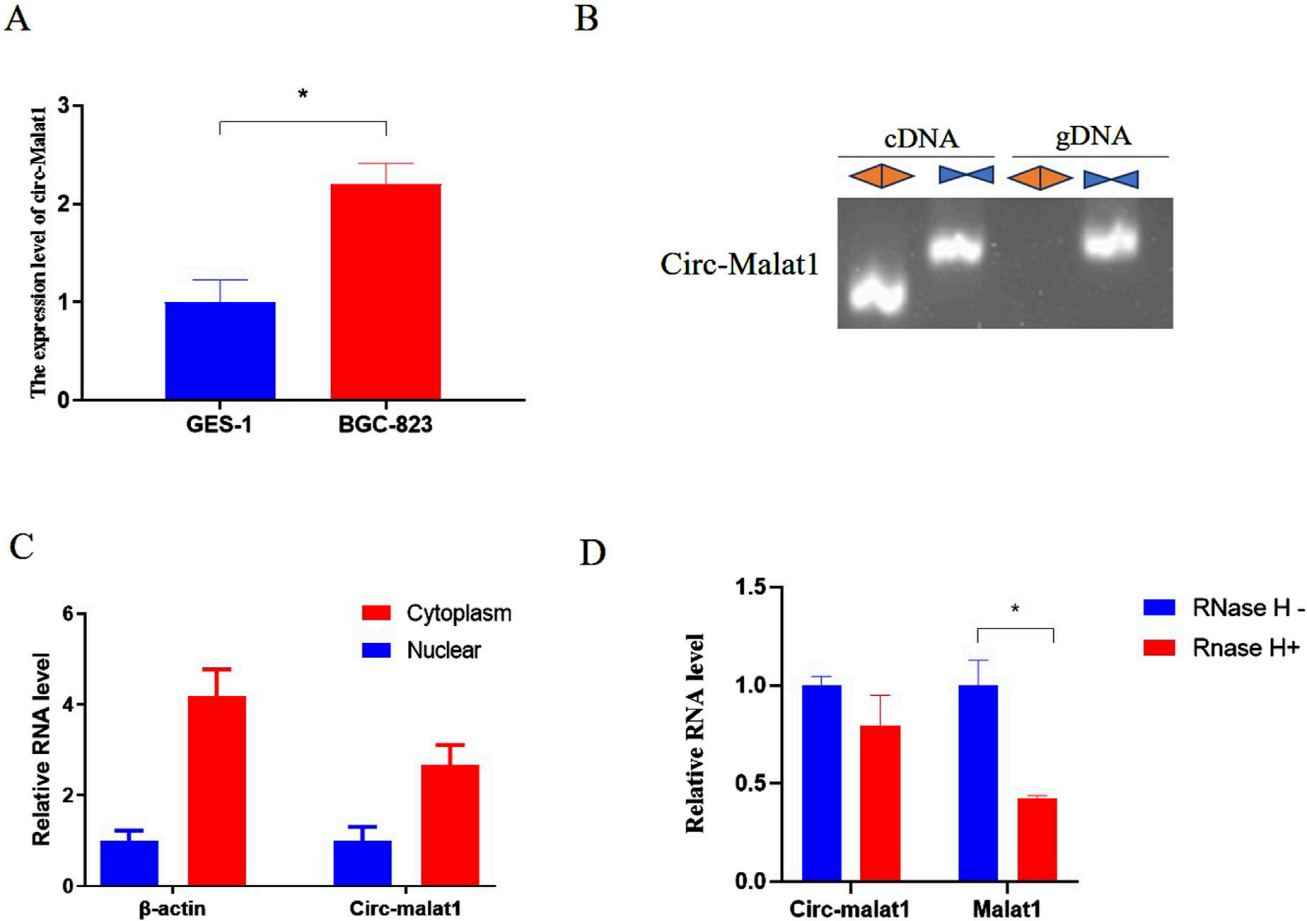
The expression characteristics of circ-malat1 in gastric cancer cells. **(A)** Detection of circ-malat1 expression in GSE-1 (normal gastric epithelial cells) and BGC-823 (gastric cancer cell) using real-time quantitative PCR, β-actin gene acts as a reference gene. **(B)** Divergent primers amplified circ-malat1 in cDNA but not in genomic DNA (gDNA). In contrast, convergent primers were able to amplify circ-malat1 in both cDNA and gDNA. **(C)** PCR was used to detect the expression of circ-malat1 in the nucleoplasm of BGC-823 cells, with β-actin serving as the reference. **(D)** After RNase H treatment, real-time quantitative PCR was conducted to detect the expression levels of circ-malat1 and malat1, CircHIPK3 acts as a reference gene. Data are presented as the mean ± SEM. *p < 0.05, n ≥ 3.

In this study, we compared the expression levels of circ-malat1 in gastric cancer cells and normal cells. We found that the expression level of circ-malat1 was significantly higher in gastric cancer cells compared to normal cells (Figure 1A). To identify endogenous circ-malat1, we designed convergent primers located within the circRNA sequence and a divergent primer pair spanning the back-splice junction of the circRNA. This design ensures the specific amplification of the circular form of the RNA, distinguishing it from the linear counterpart (Figure 1B). RNA was isolated and extracted from the nucleoplasm to detect circRNA expression, and circ-malat1 was found to be mainly expressed in the cytoplasm (Figure 1C). After treating the RNA with RNase R, the expression of linear RNA was significantly reduced, while circ-malat1 remained stably expressed (Figure 1D). This result suggests that circ-malat1 may play an important role in gastric cancer.

### Circ-malat1 promotes gastric cancer cell growth

**FIGURE 2:**
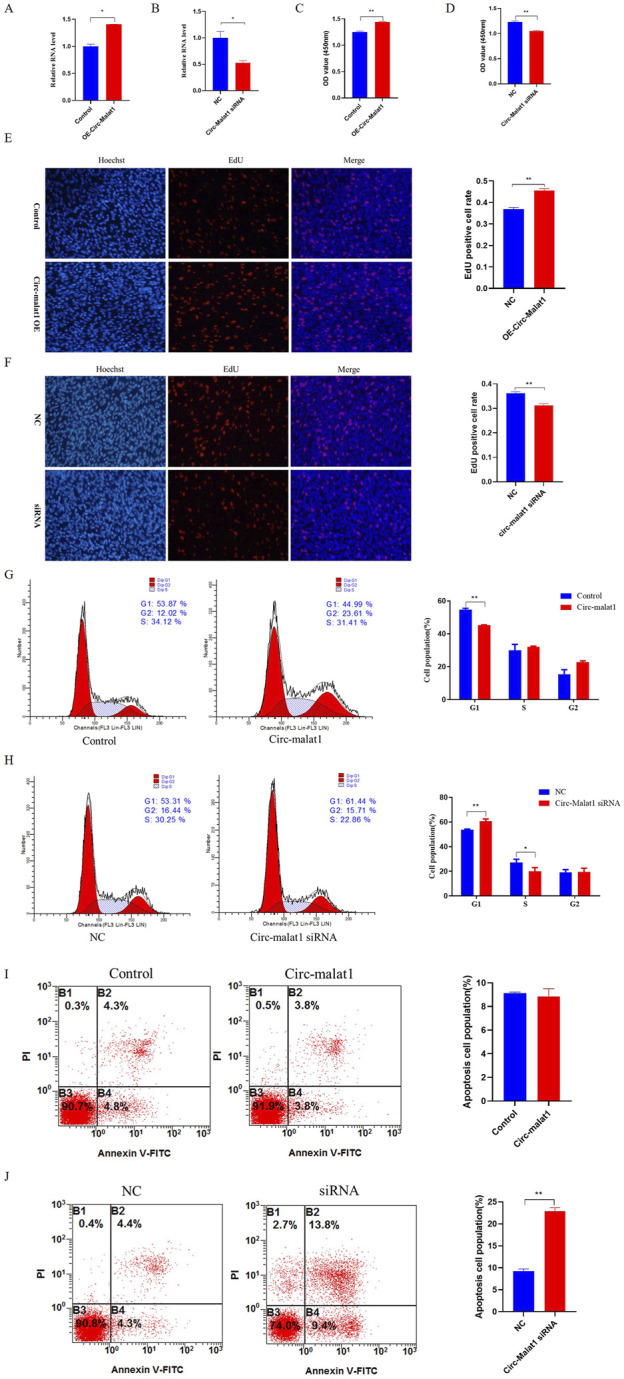
Circ-malat1 regulates gastric cancer cell growth **(A)** Detection of the overexpression efficiency of circ-malat1. **(B)** Detection of interference efficiency of circ-malat1. **(C)** After overexpressing circ-malat1, cell proliferation activity was assessed using the CCK-8 assay. **(D)** After interfering with circ-malat1 siRNA, cell proliferation activity was measured using the CCK-8 assay. **(E)** After overexpressing circ-malat1, EdU was used to assess cell proliferation. **(F)** After interfering with circ-malat1 siRNA, EdU was used to assess cell proliferation. **(G)** Cell cycle analysis was performed using flow cytometry after the overexpression of circ-malat1. **(H)**. Cell cycle analysis was conducted using flow cytometry following the interference with Circ-malat1. **(I)** Analysis of cell apoptosis by flow cytometry following the overexpression of circ-malat1. **(J)** Analysis of cell apoptosis using flow cytometry after interfering with circ-malat1. Data are presented as the mean ± SEM. *p < 0.05, **p < 0.01, n ≥ 3.

Research has shown that the expression level of circ-malat1 is significantly higher in gastric cancer cells compared to normal gastric epithelial cells. To further investigate the role of circ-malat1 in the growth of gastric cancer cells, several functional experiments were conducted. After transfection with the circ-malat1 overexpression vector or siRNA for 24 h, the efficiency of circ-malat1 expression was assessed using qRT-PCR (Figures 2A,B). Using CCK-8, it was found that overexpressing circ-malat1 exhibited a significantly faster proliferation rate of gastric cancer cells (Figure 2C). In contrast, knocking down circ-malat1 expression significantly reduced the proliferation capacity of gastric cancer cells (Figure 2D). EdU results demonstrated that overexpression of circ-malat1 increased the number of S phase cells (Figure 2E), while knockdown of circ-malat1 reduced the number of S phase cells (Figure 2F). The cell cycle assay showed that overexpression of circ-malat1 decreased the number of G1 phase cells (Figure 2G) whereas knockdown of circ-malat1 increased the number of G1 phase cells and reduced the S phase population (Figure 2H). Flow cytometry analysis of apoptosis rates revealed that overexpression of circ-malat1 has no significant effect on the apoptosis of gastric cancer cells (Figure 2I), while knockdown of circ-malat1 promoted apoptosis (Figure 2J). This finding underscores the crucial role of circ-malat1 in enhancing gastric cancer cell survival.

### Circ-malat1 acts as a competing endogenous RNA

**FIGURE 3:**
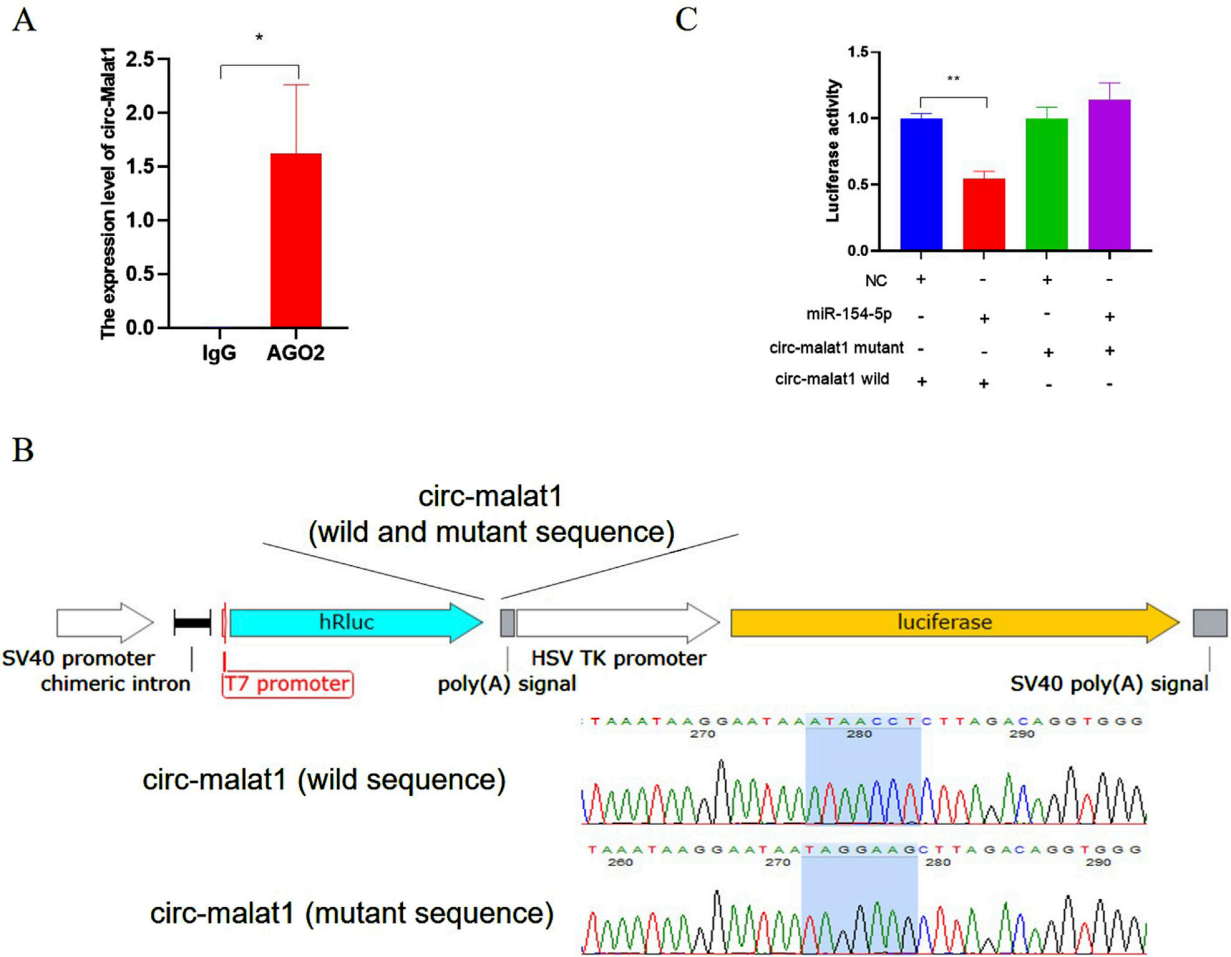
Circ-malat1 acts as a competing endogenous RNA **(A)** RNA immunoprecipitation (RIP) analyzes the binding of AGO2 to circ-malat1, using IgG as a negative control. **(B)** Construction of the psi-Check2 dual-luciferase vector containing wild-type or mutant sequences of circ-malat1. **(C)** After co-transfecting cells with the miR-154-5p mimic and the circ-malat1 dual-luciferase reporter vector (either wild type or mutant), dual-luciferase activity analysis was performed, and NC stands for miRNA negative control. Data are presented as the mean ± SEM. *p < 0.05, **p < 0.01, n ≥ 3.

The expression pattern of circ-malat1 in cells was analyzed using nuclear-cytoplasmic fractionation, revealing its predominant localization in the cytoplasm (Figure 1C). The hypothesis suggests that circ-malat1 might function as a competitive endogenous RNA (ceRNA) based on the mechanism of circRNA action. The bioinformatics predictions dataindicates the presence of 81 miRNAs binding sites in circ-malat1 (Supplementary Table S3). To further validate that circ-malat1 functions as a ceRNA, RNA immunoprecipitation (RIP) revealed enrichment of circ-malat1 by the AGO2 protein compared to the IgG group (Figure 3A). To confirm the direct binding and sequestration of specific miRNAs by circ-malat1, we constructed luciferase reporter vectors containing either the wild-type or mutant circ-malat1 sequences, in which the miR-154-5p seed sequence binding sites were mutated(Figure 3B). Based on the candidate miRNA function in gastric cancer, we selected miR-154-5p for further research (Song et al., 2018). miR-154-5p mimic and luciferase reporter vectors were co-transfected into cells, and the relative luciferase activity data are presented in Figure 3C. Compared with the control group, miR-154-5p overexpression significantly decreased wild-type luciferase activity, while it had no noticeable effect on the activity of the mutant-type luciferase. These findings collectively suggest that circ-malat1 functions as a ceRNA by competitively binding to miR-154-5p.

### Circ-malat1 regulates gastric carcinogenesis through the miR-154-5p/CCND2 axis

**FIGURE 4:**
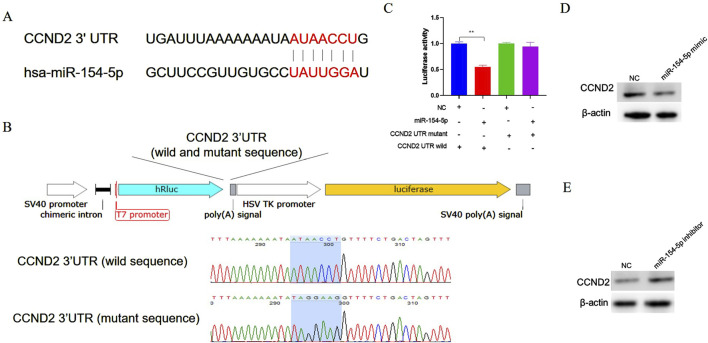
CCND2 is a target gene of miR-154-5p in gastric cancer cells. **(A)** The bioinformatics software predicts that the seed region sequence of miR-154-5p can target the 3′UTR region of the gene CCND2. **(B)** Construct a dual-luciferase reporter vector incorporating either the wild-type or mutant sequence of the 3′untranslated region (UTR) of the CCND2 gene. **(C)** After co-transfecting cells with the miR-154-5p mimic and the CCND2 gene dual-luciferase reporter vector (either wild type or mutant), dual-luciferase activity analysis was performed. **(D)** After overexpressing miR-154-5p in gastric cancer cells, the expression level of CCND2 was measured using Western blot analysis. **(E)** After inhibiting miR-154-5p in gastric cancer cells, the expression level of CCND2 was measured using Western blot analysis. Data are presented as the mean ± SEM. **p < 0.01, n ≥ 3.

Bioinformatics tool (TargetScan) was used to predict the binding sites of miR-154-5p. The prediction results indicate that there are binding sites for miR-154-5p in the 3′UTR of CCND2 mRNA (Figure 4A). Previous studies have demonstrated that disrupting CCND2 expression can inhibit the proliferation of gastric cancer cells, which heightens the significance and relevance of our research (Zhang et al., 2013). To validate the direct interaction between miR-154-5p and the 3′UTR of CCND2, the 3′UTR of CCND2 containing the predicted miR-154-5p binding sites (wild type or mutant type) were inserted into a luciferase reporter plasmid, respectively (Figure 4B). miR-154-5p mimic and luciferase reporter plasmid were co-transfected into gastric cancer cells. The results showed that co-transfected with miR-154-5p mimic and wild-type CCND2 3′UTR reduced luciferase activity, but not with the mutant 3′UTR (Figure 4C). To further validate the relationship miR-154-5p with CCND2, CCND2 expression at mRNA and protein levels was detected after transfection of miR-154-5p mimic or inhibitor. The results showed that overexpression of miR-154-5p decreased the CCND2 protein level (Figure 4D). However, the knockdown of miR-154-5p increased the CCND2 protein level (Figure 4E). The relationship between miR-154-5p and CCND2 in gastric cancer was further elucidated.

**FIGURE 5:**
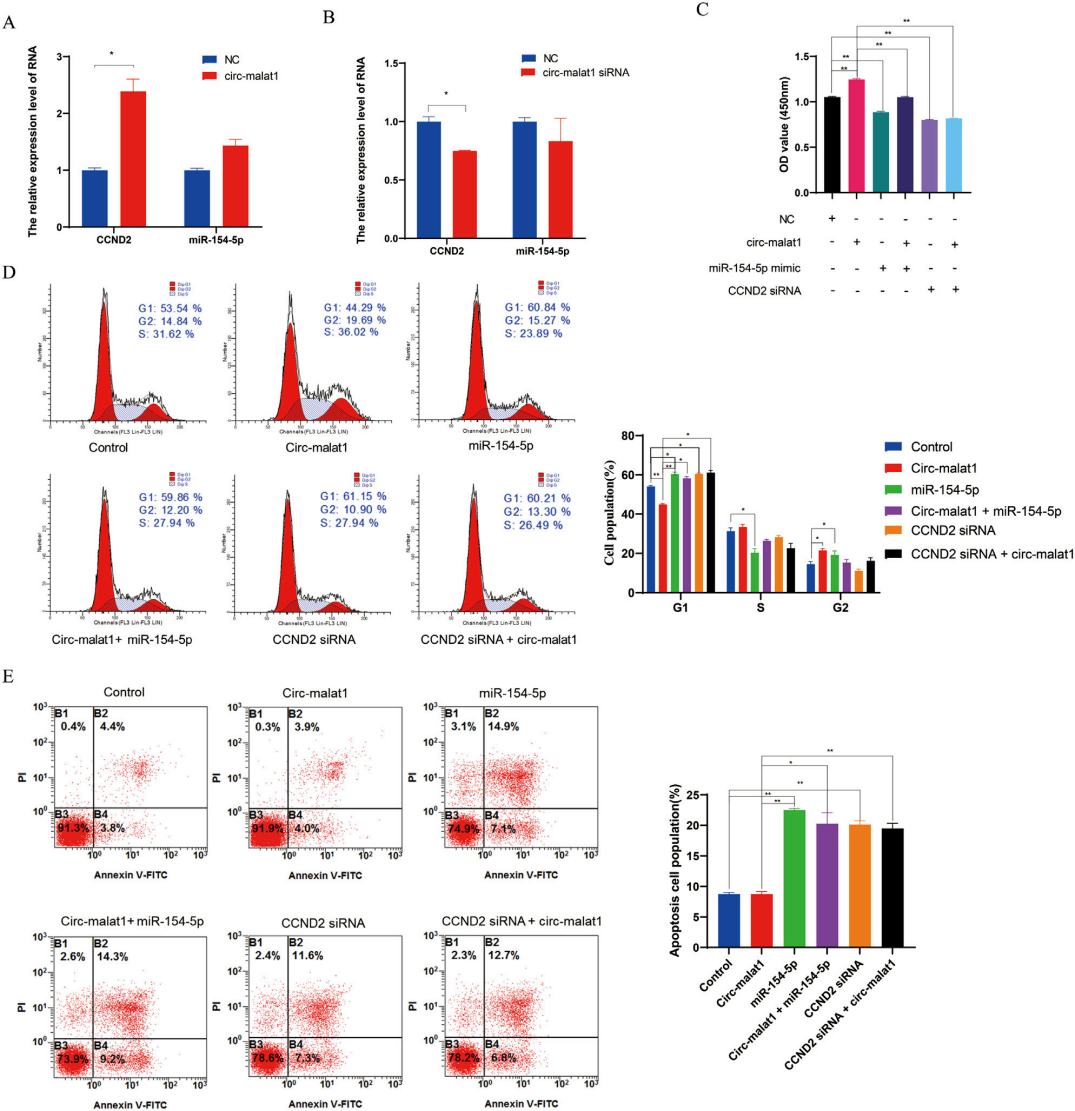
Circ-malat1 regulates gastric carcinogenesis through the miR-154-5p/CCND2 axis. **(A)** Detection of CCND2 mRNA expression levels by real-time quantitative PCR after overexpression of Circ-malat1 in gastric cancer cells. **(B)** Detection of CCND2 mRNA expression levels by real-time quantitative PCR following the interference with Circ-malat1 expression in gastric cancer cells. **(C)** CCK-8 was used to detect the effects on gastric cancer cell proliferation from overexpressing circ-malat1, overexpressing miR-154-5p, co-expressing miR-154-5p and circ-malat1, interfering with CCND2, and overexpressing circ-malat1 while also interfering with CCND2. **(D)** Flow cytometry was used to detect the effects on the cell cycle of overexpressing circ-malat1, overexpressing miR-154-5p, co-expressing miR-154-5p and circ-malat1, interfering with CCND2, and overexpressing circ-malat1 while simultaneously interfering with CCND2. **(E)** Flow cytometry was used to assess the effects on cell apoptosis following the overexpression of circ-malat1, the overexpression of miR-154-5p, the co-expression of miR-154-5p and circ-malat1, the interference with CCND2, and the overexpression of circ-malat1 while interfering with CCND2. Data are presented as the mean ± SEM. *p < 0.05, **p < 0.01, n ≥ 3.

To validate whether circ-malat1 regulates gastric cancer development through the miR-154-5p/CCND2 axis, overexpression or knockdown of circ-malat1 was performed, followed by detection of miR-154-5p or CCND2 expression using qRT-PCR. Overexpression of circ-malat1 does not affect the RNA level of miR-154-5p but promotes the mRNA level of CCND2 (Figure 5A). Conversely, the knockdown of circ-malat1 does not affect the RNA level of miR-154-5p while inhibiting the mRNA level of CCND2 (Figure 5B). To further validate the relationship between circ-malat1 and miR-154-5p/CCND2 in the regulation of gastric carcinogenesis. Compared to the circ-malat1 overexpression group, co-overexpression of circ-malat1 and miR-154-5p revealed that the proliferation ability of gastric cancer cells was inhibited, as shown by the CCK-8 assay (Figure 5C). Flow cytometry results indicated G_1_ phase cell cycle arrest (Figure 5D) and an increase in the number of apoptotic cells (Figure 5E). Furthermore, co-overexpression of Circ-malat1 and interference with CCND2 siRNA inhibited the proliferation of gastric cancer cells compared to the circ-malat1 overexpression group (Figure 5C). There was an increase in G_1_ phase cells (Figure 5D) and an increase in the number of apoptotic cells (Figure 5E). These results indicate that circ-malat1 regulates gastric carcinogenesis through the miR-154-5p/CCND2 axis.

## Discussion

Studies have shown that the expression level of circ-malat1 is significantly higher in gastric cancer cells compared to normal cells. Through a series of functional experiments, it was found that overexpression of circ-malat1 significantly promotes the proliferation of gastric cancer cells. Conversely, knocking down circ-malat1 expression inhibited the growth of these cancer cells. These results indicate that circ-malat1 plays a crucial role in the occurrence and development of gastric cancer, possibly by regulating related signaling pathways and gene expression to promote malignant behaviors in cancer cells. Therefore, circ-malat1 may be a potential therapeutic target for gastric cancer.

At present, there have been relevant research reports on circ-malat1 in other types of cancer. In colorectal cancer, circ-malat1, as a competitive endogenous RNA, enhances KAT6B expression by sponging miR-506-3p, thereby promoting cell growth, migration, and epithelial-mesenchymal transition (EMT) (Yang et al., 2023). In breast cancer progression, the knockdown of circ_0002082 (circ-malat1) enhances apoptosis in breast cancer cells and suppresses growth and metastasis by abolishing miR-508-3p, which subsequently upregulates the expression of Centromere Protein F (CENPF) (Liu et al., 2022). In hepatocellular cancer stem cells, in addition to acting as a competitive endogenous RNA that sequesters miR-6887-3p, circ-malat1 also plays a role as a translational brake of PAX5 mRNA in a novel regulatory mechanism (Chen et al., 2020). In this process, circ-malat1 forms a ternary complex with ribosomes and PAX5, thereby hindering the translation process (Chen et al., 2020). These studies suggest that circ-malat1 has the potential to serve as a molecular target in tumorigenesis. In the current study, we found that circ-malat1 is highly expressed in gastric cancer cells, and to our knowledge, no relevant research has been reported, sparking our interest in further exploration. Our results showed that the overexpression of circ-malat1 promotes the growth of gastric cancer cells while inhibiting circ-malat1 expression suppresses these abilities. This is consistent with the role of circ-malat1 in other tumorigenesis processes (Liu et al., 2022; Chen et al., 2020).

In recent years, circRNA has emerged as a research hotspot in gastric cancer (Li et al., 2020), with its functions being widely reported across various biological processes, including tumor growth (Zhang et al., 2021), apoptosis (Peng et al., 2020), metastasis (Shen et al., 2023), and invasion (Ding et al., 2024). However, as research techniques have advanced, understanding its molecular mechanisms in detail has become a crucial focus of study. Research has shown that circRNA’s molecular functions are closely tied to its subcellular localization. When distributed in the nucleus, circRNA can bind to proteins and regulate gene transcription or alternative splicing. circURI1 directly interacts with heterogeneous nuclear ribonucleoprotein M (hnRNPM) to modulate alternative splicing of genes, involved in the process of cell migration, thus suppressing gastric cancer metastasis (Wang X. et al., 2021). CircSLC22A23 promotes gastric cancer cell proliferation, migration, and invasion by participating in the regulation of epidermal growth factor receptor (EGFR) transcription through the activation of HNRNPU protein expression (Wu et al., 2024). When localized in the cytoplasm, circRNA can similarly bind to proteins and influence mRNA translation. Additionally, circRNA can function as a molecular sponge, competitively binding to microRNAs and thereby affecting the expression of downstream genes. CircNRIP1 functions as a molecular sponge, competitively binding to miR-149-5p, thereby promoting gastric cancer progression by influencing the expression level of AKT1 (Zhang et al., 2019). In addition, certain circRNAs are expressed in both the nucleus and the cytoplasm. Circ_SMAD4 promotes gastric cancer tumorigenesis by recruiting TCF4 to facilitate CTNNB1 transcription in the nucleus and sequestering miR-1276 in the cytoplasm to prevent the silencing of CTNNB1 mRNA, thereby activating the Wnt/β-catenin pathway (Wang L. et al., 2021). For relevant research on the regulation of gastric cancer by circRNA, please refer to the review article (Ma et al., 2024). In this study, we found that circ-malat1 is primarily expressed in the cytoplasm. In line with their mode of action, circ-malat1 can function as molecular sponges, competitively binding to miR-154-5p to regulate CCND2 gene expression. In previous studies, miR-154-5p was found to inhibit the growth and invasion of gastric cancer cells by targeting the DIXDC1/WNT signaling pathway (Song et al., 2018). Besides, according to previous studies (Chen et al., 2020), this circular RNA may also have other mechanisms of action in gastric cancer, which require further investigation.

In conclusion, current research has revealed that circ-malat1 functions as a competitive endogenous RNA by adsorbing miR-154-5p, thereby promoting the growth of gastric cancer cells. So, these studies further elucidate the potential of circ-malat1 as a molecular target for therapeutic intervention in gastric cancer.

## Data Availability

The original contributions presented in the study are included in the article/Supplementary Material, further inquiries can be directed to the corresponding author.
